# Comparison between two FISH techniques in the *in vitro* study of cytogenetic markers for low-dose X-ray exposure in human primary fibroblasts

**DOI:** 10.3389/fgene.2013.00141

**Published:** 2013-07-29

**Authors:** D. Nieri, F. Berardinelli, A. Antoccia, C. Tanzarella, Antonella Sgura

**Affiliations:** Department of Sciences, Roma Tre UniversityRoma, Italy

**Keywords:** marker of ionizing radiation exposure, biodosimetry of ionizing radiations, chromosome aberrations, mFISH, hyper-radiosensitivity

## Abstract

This work is about the setup of an *in vitro* system to report low-dose of X-rays as measured as cytogenetic damage. Q- and multicolor FISH (m-FISH), for telomere length and chromosome instability analysis, respectively, were compared to evaluate their sensitivity in the low-dose range in human primary fibroblasts. No telomere length modulation was observed up to 1 Gy in cycling fibroblasts, though reported for high doses, by that frustrating the purpose of using it as a low-exposure marker. To date the m-FISH is the best setup for the assessment of the chromosome structural damage: it allows stable and instable aberrations to be detected all over the karyotype. Stable ones such as balanced translocations, are not eliminated due to cell-cycle as unstable ones, so they are considered transmissible markers for retrospective dosimetry. The induction of chromosome damage showed a clear dependence on dose delivered; unstable aberrations were demonstrated after doses of 0.1 Gy, and stable aberrations after doses higher than 0.5 Gy. Summarizing, q-FISH is unfit to report low exposures while m-FISH provides better results: unstable aberrations are sensible short-term reporters, while stable ones long report exposures but with a higher induction threshold.

## Introduction

The use of ionizing radiations (IR) in many fields of human activities has promoted the need to develop radiation protection. In radiation protection, the prerequisite for limiting exposure, minimizing secondary effects, and applying the protection procedures, is the quantification of the exposure.

Exposed workers are equipped with physical dosimeters allowing for the qualified experts to assess the dose for each worker or to control the workplace, but in case of incidental or medical exposure, there is even no possibility to use physical dosimeters. In some cases, the biological dosimetry is the only way to get information about the IR exposure (Bauchinger et al., 2001). The observation of biological markers that can be related to the adsorbed dose can play an important role in study of IR-related health risk, and in this respect the identification of the most appropriate biomarkers is essential. Among these, cytogenetic endpoints are considered good biomarkers because of their high degree of specificity and sensitivity (IAEA, 2011). For example, the dicentric chromosomes is the biomarker of choice for investigating recent IR exposure (Bauchinger et al., 1984; Natarajan, 2002). In this context, it has been demonstrated that the dicentric assay is able to assess health risks and guide medical treatment decisions in the event of large scale radiation accidents like Chernobyl (Piatkin et al., 1989) or Goiania (Ramalho and Nascimento, 1991). As a consequence of an acute exposure, when a blood sample for chromosome analysis must be obtained as soon as possible, the dicentric assay represents the method of choice. In fact, the analysis of dicentrics (unstable aberrations) in solid-stained chromosome preparations is very reliable for the evaluation of recent and acute radiation exposures, although not for chronic or past exposures in that the yield of dicentric chromosomes decreases over the time after irradiation (Bauchinger, 1995). FISH using whole human chromosome-specific DNA probes (Chromosome Painting) has opened the way to new possibilities for detecting stable aberrations, such as balanced translocations, and nowadays it is widely used for biological dosimetry of IR (Tucker, 2001; Camparoto et al., 2003; Tucker and Luckinbill, 2011). In fact, as the aberrations involving the painted chromosomes represent only a subset of the total aberrations, the higher is the number of stained chromosome pairs in the same metaphase, the higher is the supplied information.

In this respect, the development of multicolor FISH (m-FISH) allowed all homolog pairs to be differentiated and the whole genome to be analyzed (Speicher et al., 1996). This implementation has greatly improved the ability to identify chromosome aberrations and the capability to predict the fate of exposed cells or individuals.

Unfortunately, other relevant cytogenetic endpoints have been so far less investigated, and to the best of our knowledge poor analysis has been carried out on the effect of the low-dose range of IR on the telomeric functions though data reported in the literature infer a relationship between IR exposure and telomere length (Ducray and Sabatier, 1998). Telomeres are specialized nucleoprotein complexes that serve as protective caps of linear eukaryotic chromosomes. Loss of telomere function can lead to genomic instability and cancer progression (Blasco et al., 1997) and is associated with radiation-induced genomic instability, increased radiation sensitivity (Berardinelli et al., 2012), loss of cellular viability and senescence (Blasco, 2005).

IR can harm the telomere by the direct break of the telomeric DNA strand (Bolzan and Bianchi, 2006), or through the oxidation to 8-oxodG of the deoxyguanosine of the sequence TTAGGG (von Zglinicki et al., 2000). Moreover it should be considered that telomere is repaired less efficiently than the rest of the genome (Opresko et al., 2005). Other works have demonstrated a telomere elongation, both telomerase-dependent in lymphoblasts (Hande et al., 1998; Neuhof et al., 2001), and telomerase-independent in fibroblasts (Berardinelli et al., 2010). Far from being clarified, the consequences of the IR exposure on the telomere homeostasis have been studied for a long time with conflicting results (Ducray and Sabatier, 1998; Hande et al., 1998; Neuhof et al., 2001; Schuck et al., 2002), and the meaning of such telomere length variations has not been clarified as yet.

These results seem to indicate the telomere as a potential exposure biomarker, but further studies are needed to validate the use of this endpoint especially for the low-dose range.

In recent years, a great deal of attention has been devoted to the study of biological effects of low-dose of IR for their relevance in radiation protection of many different contests as screening tests, environmental and occupational exposures, frequent-flyer risks, manned space exploration, and so on. In this work, our interest was to check the sensitivity of the aforementioned endpoints (karyotype damage and telomere length) to low doses of X-rays.

To the best of our knowledge, neither studies on telomere homeostasis nor m-FISH analysis have been carried out so far in human primary fibroblasts in the dose range between 0 and 1 Gy of X-rays, though telomere homeostasis alteration and genomic instability have been demonstrated in HFFF2 human primary fibroblasts exposed to high doses of X-rays and protons (Berardinelli et al., 2011).

Therefore, to investigate in details telomere homeostasis and chromosome alteration induction as a function of the dose, AG01522 human primary fibroblasts were exposed to low-doses of X-rays, and tested for telomere length modulation as well as for chromosome damage.

## Materials and methods

### Cell lines and culture conditions

Human primary fibroblasts AG01522 (Coriell Institute, Camden, NJ, USA) were cultured in EMEM medium (Euroclone, Pero, Italy) supplemented with 15% fetal bovine serum (Euroclone, Pero, Italy), 100 units/ml penicillin, 100 mg/ml streptomycin, and 2 mM L-glutamine, 1% non-essential aminoacids and grown in 5% CO_2_ atmosphere at 37°C. In these conditions, the cell doubling time, evaluated from the growth curves, was 22 ± 1 h. Cells used in the present work were at the 28th population doubling.

### Irradiation procedures

For X-irradiation, cells seeded in plastic petri dishes, were irradiated by a Gilardoni apparatus (250 KV, 6 mA, dose-rate 0.53 Gy/min) with 0.1, 0.25, 0.5, and 1 Gy. Sham irradiated cells were used in all the experiments as control cells. For each experiment, cells were seeded 48 h before irradiation.

### Collection of chromosome spreads

Chromosome spreads were obtained following 30 min incubation in Calyculin-A (30 μM; Wako, Japan), a phosphatase inhibitor, which induces chromosome condensation irrespectively of cell-cycle phase (Durante et al., 1998). In this work only G_2_ prematurely condensed (PCC) chromosomes and metaphasic (M) chromosomes were scored by the cytogenetic analysis. PCC and M chromosomes were collected after a treatment with hypotonic KCl (75 mM) for 30 min at 37°C, followed by fixation in freshly prepared Carnoy solution (3:1 v/v methanol/acetic acid). The cell suspension was dropped onto slides and utilized for cytogenetic analysis.

### Telomeric quantitative fluorescent *in situ* hybridization (q-FISH)

**Figure 1:**
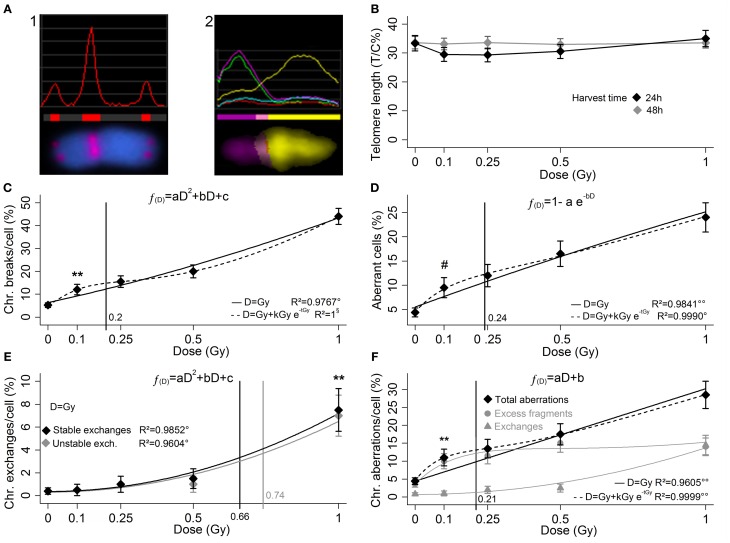
**In the q-FISH analysis for telomere length (A1) the telomeric signal is compared to the signal of the chromosome 2 centromere, stained with the same fluorophore**. In the m-FISH **(A2)** each chromosome has a specific multi-fluorophore staining which allows the structural aberrations to be detected all over the karyotype. Here the image analysis of aberrant chromosome (1′ 15). **(B)** Up to 1 Gy no telomere length variation is reported 24 and 48 h after a low doses of X-rays, and no threshold dose for telomere length variation is determinable. **(C)** Yield of radio-induced chromosome breaks (LQ fit), **(D)** aberrant cell fraction (exponential saturation fit), **(E)** yield of stable and unstable exchanges (LQ fits, black and gray markers and curves, respectively), and yield of total aberrations **(F)** (linear fit), as sum of excess fragments and exchanges (gray circles and triangles, respectively). The curves represents the regressions with and without introducing the low-doses HRS correction (dashed and solid, respectively). For each cytogenetic damage type the vertical line represents the 95% upper confidence level of the calculated dose that induces a detectable damage yield (reported in Table 1). The markers (^*^, ^#^) indicate the first experimental points significantly beyond the control. Bars represents the standard errors. Mann–Whitney's *u*-test, ^**^p < 1%; Fisher's exact test, ^#^*p* < 5%; Fisher's *F*-test, °*p* < 5%; °°*p* < 1%; §, no uncertainty in the regression, *p* is not calculated.

Two days after the preparation of chromosome spreads, slides were rinsed with PBS pH 7.5, and fixed in 4% formaldehyde for 2 min. After two rinses in PBS, the slides were incubated in pepsin solution for 10 min, rinsed, and dehydrated through graded ethanols. Slides and probes (Cy3-linked telomeric and chromosome 2 centromeric PNA probe, DAKO Cytomation, Denmark) were co-denatured at 80°C for 3 min and hybridized for 2 h at room temperature in a humidified chamber. After hybridization, slides were washed twice with 70% formamide, 10 mM Tris pH 7.2 and 0.1% BSA for 15 min, followed by three washes in TBS (0.1 M Tris pH 7.5, 0.15 M NaCl) and 0.08% Tween20 for 5 min each. Slides were then dehydrated with an ethanol series and air dried. Finally, slides were counterstained with DAPI. Images were captured at 63 × magnification with the Axio Imager M1 microscope (Carl Zeiss, Jena, Germany), and the telomere size was analyzed with ISIS software (MetaSystems, Altlussheim, Germany). The software calculates telomere lengths as the ratio between the fluorescence of each telomere and the fluorescence of the centromere of chromosome 2 (T/C), used as the internal reference in each metaphase analyzed (Figure 1A). Centromere 2 sequence has a stable length to be used as internal reference (Perner et al., 2003). Data are expressed as a T/C%. At least 1800 chromosomes were analyzed for each experimental point in two different experiments.

### Multicolor fluorescent *in situ* hybridization (m-FISH)

Fixed cells were dropped onto glass slides and hybridized with the 24XCyte Human Multicolor FISH Probe Kit (MetaSystems, Altlussheim, Germany), following the manufacturer's instructions. Briefly, slides were denatured in 0.07 N NaOH and then rinsed in graded ethanols. Meanwhile, the probe mix was denatured in a MJ mini personal thermal cycler (Bio-Rad laboratories, Hercules, CA, USA) with the following program: 5 min 75°C, 30 s 10°C, and 30 min 37°C. Samples were then hybridized in a humidified chamber at 37°C for 48 h, followed by one wash in saline-sodium citrate (SSC) buffer for 5 min at 75°C and counterstaining with DAPI. Finally, metaphases were visualized and captured using an Axio-Imager M1 microscope. Karyotyping and cytogenetic analysis of each single chromosome was performed by means of the ISIS software. Two hundred metaphase spreads were analyzed for each experimental point in two different experiments, and 500 metaphases for sham-irradiated control.

Each chromosome of a metaphase spread was examined on the basis of its unique fluorochrome profile: in Figure 1A2 is shown an aberrant chromosome (1′ 11) as detected by image analysis. Structural chromosome aberrations were classified following the mPAINT system (Cornforth, 2001). Aberrations were classified as excess acentric fragments (i.e., fragments not associated with an exchange), stable exchanges (i.e., balanced translocations), and unstable exchanges (i.e., dicentrics, centric rings, and unbalanced translocations).

## Results

### q-FISH analysis for telomere length study

This q-FISH investigation on telomere length variation was undertaken to check the possibility of plotting a dose-response curve in the low-dose range (0, 0.1, 0.25, 0.5, and 1 Gy). A dataset has been already collected about the effect of IR on the telomere length in other fibroblast (HFFF2) (Berardinelli et al., 2011), and in our opinion the normal fibroblasts are a good model because they are telomerase-negative cells, so that at least this activity does not interfere in the response.

Q-FISH analysis has been performed at two harvest times, in two different experiments, in order to check any telomere modulation 24 h after exposure, as well as some possible delayed effect at 48 h.

The measurements of the telomere length in sham-irradiated samples display the T/C% ratio value approximately of 33%. Data show only an elusive shortening 24 h after irradiation, apparently non-dose-dependent and moreover unconfirmed at 48 h (Figure 1B). This inconclusive result allows us to conclude that telomere length measurement with the q-FISH is an unsuitable approach to report low-dose exposures.

### m-FISH for chromosome instability analysis

Unstable aberrations (i.e., acentric fragments and unstable exchanges), are known to be largely lost during mitotic anaphase, so that their persistence in the cells decays with time in few cell generations (Bauchinger, 1995; Natarajan, 2002). Conversely the stable exchanges (i.e., balanced translocations), are maintained in the subsequent generations (Tucker and Luckinbill, 2011) and are detectable in the injured cell progeny even in case of highly-cycling cells, such as spermatogonial stem cells, for at least 1 year (Donner et al., 2010). Both stable and unstable aberrations can be markers of recent exposure, but only the stable ones can long report the chromosomal radio-induced damage (RID). Also in non-cycling cells, such as peripheral blood lymphocytes, their half-life is much longer and is measured in years, while the half-life of unstable exchanges in months (Fucić et al., 2007). It is important to get a reliable basal value for both stable and unstable aberrations: any increase in aberration frequency is suitable to report an adsorbed dose, as much as the spontaneous aberration frequency value is characterized with the least possible uncertainty. With this purpose, 500 control metaphase spreads have been analyzed to be compared with a set of samples irradiated with doses of X-rays up to 1Gy (200 metaphase spreads per dose in two independent experiments).

Data are presented in Table 1 and show a clear dose-dependent damage yield. Five parameter have been considered, breaks, aberrant cells fraction, exchanges (stable and unstable), and total aberrations. Among these, the total aberrations (sum of excess fragments and exchanges, fragments are the larger part) and unstable exchanges report only recent exposures because of their rapid decay, and consequently also the aberrant cell fraction parameter, while stable exchanges long report RID. IR lead to chromosomal breaks and, in turn, breaks produce acentric fragments and chromosomal exchanges. The break number is the sum of chromosome breaks necessary and sufficient to give the structural aberrations observed. A cell is considered aberrant if carrying any structural chromosome aberration (note that 4.4% of the control cells carries some kind of aberration).

**Table 1:** **Results of m-FISH analysis in irradiated AG01522 fibroblasts: scores and relative frequencies**.

**Dose (Gy)**	**Cells scored**	**Breaks (%)**	**Aberrant cells (%)**	**Stable exchanges (%)**	**Unstable exchanges (%)**	**Total aberrations (%)**
0	500	26 (5.2)	22 (4.4)	2 (<0.5)	2 (<0.5)	22 (4.4)
0.1	200	24 (12.0)^**^	**19 (9.5)^#^**	1 (0.5)	1 (0.5)	22 (11.0)^**^
0.25	200	31 (15.5)	24 (12.0)	2 (1.0)	2 (1.0)	27 (13.5)
0.5	200	40 (20.0)	33 (16.5)	3 (1.5)	2 (1.0)	35 (17.5)
1	200	88 (44.0)	48 (24.0)	15 (7.5)^**^	14 (7.0)^**^	57 (28.5)
			Calculated sensitivity threshold dose [Gy] (95% upper CI)		
		**0.11 (0.20)**	**0.12 (0.24)**	**0.53 (0.66)**	**0.60 (0.74)**	**0.12 (0.21)**

The first point significantly beyond the control (in bold and marked), empirically determinates the least dose that produces a significant effect (p < 5%) on the header parameter (markers omitted for the following points). At the bottom is reported the least theoretical dose that produces an observable effect as calculated on the basis of the non-HRS-corrected fitted functions (see below in the text). Mann–Whitney's u-test,

** *p* < 1%; Fisher's exact test,

# *p* < 5%.

The analysis of the chromosome breaks yield (Figure 1C) shows a linear-quadratic (LQ) fit very close to the linearity. At 0.1 Gy is evident a significant increase beyond the control of the chromosome break frequency (*p* < 1%). The fraction of cells with karyotype with structural aberrations follows an exponential-saturation model (Figure 1D), and a dose of 0.1 Gy produces a significant increase of this fraction (*p* < 5%). In this dose range the chromosome exchanges yield (i.e., stable and unstable exchanges) approximately follows a LQ fit (Figure 1E), while for high doses it is supposed to become approximately linear (Lee et al., 2011a), and the frequency increase is experimentally demonstrated in both cases only for the dose of 1 Gy (*p* < 1%). The total aberrations yield (Figure 1F) is the sum of excess fragments and total exchanges (both stable and unstable) represented in gray (circles and triangles, respectively). The total aberration parameter shows a linear increase with the dose, while at higher doses it is supposed to become LQ (Lee et al., 2011b). All the proposed regression models must be considered limited to this dose range.

As in Figures 1C–F a peak of damage excess can be recognized around 0.1–0.25 Gy, alternative low-dose hyper-radiosensitivity-like (HRS) models (Marples et al., 2004) have been successfully tested (dashed lines, irrelevant in Figure 1E and not shown for clarity). To point out that the HRS models are biased for the lack of experimental points far from the HRS region, thus the non-HRS regressions were considered preferable as well as largely more manageable for further speculations.

In fact, beside the empirical threshold dose of each parameter, also the theoretical threshold was calculated on the basis of the respective non-HRS-corrected regression function (solid curves in Figures 1C–F). The upper limit of the confidence interval (95% CI), calculated by the uncertainty of the respective regression function (Long and Xu, 2005), is reported as vertical line in Figures 1C–F with a meaning of conservative threshold.

## Discussion

This work was aimed at testing the reliability of the q-FISH and m-FISH techniques in reporting a low X-ray dose. In our experimental setting, data on telomere modulation show q-FISH as totally ineffective in the low-dose range. No threshold dose is determinable within 1 Gy, either empirical or calculated, which achieves a reliable telomere length variation. Although the possibility that radio-induction of the telomere shortening is below the technique sensibility threshold cannot be excluded, the telomere as a marker of low-dose of X-ray exposure should not be considered, at least as measured by the q-FISH.

Conversely, the m-FISH was demonstrated to be a powerful technique for the richness of information supplied by the simultaneous analysis of 23 chromosomes pairs. Among the considered parameters, breaks, aberrant cell fraction, and total aberrations are all three very sensitive parameters (empirical threshold dose for detectable damage induction 0.1 Gy for all; calculated threshold between 0.1 and 0.25 Gy for all), but unfortunately they are short-term RID reporters.

The lower sensitivity of the stable exchanges parameter (empirical and calculated threshold between 0.5 and 1 Gy) is rewarded by the fact that it shows potential as a retrospective reporter of exposures distant in time (Fucić et al., 2007; Donner et al., 2010). Note that this lower sensitivity is not due to a technique failure but to the X-ray damage induction mode: in this dose rage the exchange frequency seemed to be weak and approximately dependent on the square of the dose, and up to 0.5 Gy this kind of RID is negligible.

The unstable exchanges parameter instead is completely unfit to dosimetric purposes because the short persistence of such structural aberrations is associated with a high induction threshold (empirical and calculated threshold between 0.5 and 1 Gy). For these unsatisfactory features this parameter as a marker of low-dose of X-ray exposure should not be considered.

To note that in three cases out four the empirical thresholds are lower than the calculated thresholds. It could be attributable to the fact that the system responded to the X-rays with an excess of damage for the doses 0.1 and 0.25 Gy, recalling a possibility of a kind of low-dose HRS, and actually such an occurrence has been reported with classical non-molecular cytogenetic techniques on rodent and human cells (Tsoulou et al., 2001; Nasonova et al., 2006). Nevertheless the regression functions without low-dose HRS-like correction were preferred because without further experimental points far from HRS peak a conclusive evidence is missing, and secondly for practical reasons of calculation of the thresholds doses for the detectable damage induction.

The use of clearly low-dose hyper-radiosensitive cells should improve the system sensitivity relatively to the parameters with a threshold around the hypothetic HRS peak (i.e., chromosome breaks, aberrant cells, and total aberrations, all short-term RID reporters), in fact the calculated thresholds may be overestimated in this work. On the other hand, relatively to the parameters with an induction threshold beyond the low-dose HRS range (i.e., stable and unstable exchanges, long- and short-term RID reporters, respectively), the calculated thresholds should be similar to the observed thresholds, and it should not be profitable the use of low-dose hyper-radiosensitive cells.

In conclusion, this work is to be considered a rather successful testing of the recent molecular cytogenetic techniques to the setup of a biosensor system, though it should be pointed out that adherent growing cell lines are not systems which would let suppose a practical use for biodosimetry, however, they are well-suitable for the testing of molecular cytogenetic techniques.

In perspective, after this pilot experiment, the next step should be to carry out this feasibility study in lymphocytes, which show a higher cytogenetic damage induction and which have the best potential to biodosimetry purposes (Tucker, 2008).

### Conflict of interest statement

The authors declare that the research was conducted in the absence of any commercial or financial relationships that could be construed as a potential conflict of interest.
